# Carrier detection probabilities for autosomal recessive variants in unrelated and consanguineous couples — an evaluation of the 86 genes of the ACMG ‘Tier 3’ panel

**DOI:** 10.1007/s12687-022-00593-0

**Published:** 2022-06-04

**Authors:** Jörg Schmidtke, Michael Krawczak

**Affiliations:** 1 Medizinische Hochschule Hannover, Carl-Neuberg-Strasse 1, 30625 Hannover, Germany; 2 Institute of Medical Informatics and Statistics, Kiel University, University Medical Centre Schleswig-Holstein Campus Kiel, Brunswiker Strasse 10, 24105 Kiel, Germany; 3 Amedes MVZ Wagnerstibbe, Georgstrasse 50, 30159 Hannover, Germany

**Keywords:** Autosomal recessive disease, Carrier screening, Gene panel, Carrier detection probability, Consanguinity

## Abstract

Carrier screening for autosomal recessive variants has become a cornerstone of community and public health genetics. While the first carrier screening programs were confined to conditions with relatively high prevalence, and hence well-known carrier frequency, the number of candidate genes has increased greatly since the advent of high-throughput DNA sequencing technologies. The epidemiological database of the ensuing gene panels is mostly sparse, and judgement of their performance is, therefore, anything but straightforward. We therefore derived estimates of the carrier detection probabilities among non-consanguineous and consanguineous couples as expected using the ‘Tier 3’ carrier screening gene panel recently recommended by the American College of Medical Genetics (ACMG). For non-Finnish Europeans, the respective estimate for unrelated couples equals 0.63%, implying that the ACMG Tier 3 panel accounts for over 90% of the genetic load for autosomal recessive diseases in this population. Among the offspring of first cousins, the corresponding incidence is expected to be tenfold higher, an increase still consistent with previous estimates of the overall risk of birth defects for this type of mating. Our considerations are intended to aid the implementation of carrier screening programs and to provide additional support to reproductive counselling and to obtaining informed consent.

Carrier screening for autosomal recessive variants has become a cornerstone of community and public health genetics (Antonarakis 2019; Cornel et al. 2021). Its primary and often overlapping goals are disease prevention (Christianson and Modell 2004) and facilitation of reproductive decision-making (Henneman et al. 2016). The first carrier screening programs, which started in the 1970s, were confined to a few clinically severe conditions of high prevalence in particular ethnic groups or geographic regions such as Tay-Sachs disease in Ashkenazi Jews (Kaback et al. 1993) and beta-thalassemia in the Mediterranean (Cao et al. 1997). Even when cystic fibrosis carrier screening was introduced as the first pan-ethnic such endeavour in the 1990s (Castellani et al. 2010), the existing programs still targeted highly selected conditions so that their performance, measured in terms of carrier detection probabilities, could be inferred reliably from available epidemiological data. With the advent of high-throughput DNA sequencing technologies, however, the number of candidate genes for screening has increased greatly, and the coverage of current carrier screening programs ranges from a handful of genes (Gregg et al. 2021) via several hundred genes (Bell et al. 2011) to whole exomes (Kirk et al. 2019). The epidemiological database of these panels, however, is mostly sparse, and the judgement of their performance is anything but straightforward.

A recently published American College of Medical Genetics (ACMG) practice resource (Gregg et al. 2021) reported carrier frequencies for recessive pathogenic variants in 86 key autosomal genes (‘Tier 3 genes’), as derived from gnomAD v2.0.2 (https://gnomad.broadinstitute.org/). We evaluated this list in terms of its potential to identify at-risk couples (Schmidtke and Krawczak 2022) and concluded that the ACMG Tier 3 genes cover more than 50% of the autosomal recessive ‘genetic load’ of pairs of unrelated individuals.

Notably, the ACMG practice resource (Gregg et al. 2021) was based upon an earlier report by Guo and Gregg (2019) that contained the specific gnomAD-derived carrier frequencies for autosomal recessive variants in six different human ancestries, namely Africans (AFR), Hispanics (AMR), East Asians (EAS), South Asians (SAS), Non-Finnish Europeans (NFE) and Ashkenazi Jews (ASJ). Similar to our own analysis (Schmidtke and Krawczak 2022), Guo and Gregg (2019) also clarified that, under the assumption of stochastic independence between genes, the probability of both in an unrelated couple being simultaneous carriers of variants in at least one of *n* genes (henceforth referred to as the ‘couple carrier detection probability’) can be calculated from known gene-specific carrier frequencies, *f*_*i*_, as

1 $$1-{\prod }_{i=1}^{n}\left[1-{f}_{i}^{2}\right]$$

Briefly, $1-{f}_{i}^{2}$ equals the probability that at least one in an unrelated couple is not a mutation carrier for the *i*th gene. Owing to stochastic independence, multiplication of these terms (symbolized by capital Greek letter Π) yields the overall probability of a lack of simultaneous carriership for all genes combined i.e. the complement of the sought-for quantity. Subtracting the product from 1 hence yields the carrier detection probability for the mating type in question. It is worthy of note that, by design, formula 1 explicitly accounts for the possibility of more than one recessive disease affecting a single mating.

When formula 1 is applied to the 86 ACMG Tier 3 genes, the resulting couple carrier detection probabilities are 1.84% (AFR), 1.61% (ASJ), 0.63% (NFE), 0.32% (AMR), 0.28% (SAS) and 0.19% (EAS), respectively (see rightmost column of Table 1). Empirical estimates of the yield of an expanded carrier screening in diverse ethnic groups were derived before in a study by Haque et al. (2016) of nearly 350,000 reproductive-aged individuals, primarily from the USA. The respective samples were tested with the Family Prep Screen (Counsyl™ Inc., San Francisco) covering a large number of autosomal and X-linked recessive conditions. The resulting disease risks (see right-most column of Table 5 of the original publication) correspond well to approximately 1/4 of the couple carrier detection probabilities projected in the present study. This underpins the validity of our calculations and reflects the notable overlap between the 94 genes considered by Haque et al. (2016) and the 86 ACMG Tier 3 genes.

**Table 1:** Couple carrier detection probabilities expected for the 86 ACMG Tier 3 genes, by ancestry and degree of relatedness

Ancestry^a^	Coefficient of relatedness (*r*)^b^			
	1/4	1/8	1/32	0
AFR	10.67%	6.35%	2.98%	1.84%
ASJ	17.23%	9.74%	3.71%	1.61%
NFE	11.80%	6.37%	2.09%	0.63%
AMR	8.18%	4.33%	1.33%	0.32%
SAS	7.07%	3.73%	1.15%	0.28%
EAS	5.53%	2.89%	0.87%	0.19%

^a^*AFR* Africans, *AMR* Hispanics, *EAS* East Asians, *SAS* South Asians, *NFE* non-Finnish Europeans, *ASJ* Ashkenazi Jews

^b^1/4: uncle/aunt-niece/nephew; 1/8: 1st-degree cousins; 1/32: 2nd-degree cousins

In his 2019 review on carrier screening, Antonarakis (2019) recalled that a 30-year-old Canadian survey (Baird et al. 1988) of > 1 million neonates still represents the most comprehensive assessment of the burden of autosomal recessive diseases in a population of European descent. The incidence there was estimated to equal 1.7 in 1000 newborns, which correspond to an underlying joint carrier probability of 4∙0.17 = 0.68%. Therefore, we may surmise that the ACMG Tier 3 gene account, not only for over 50%, but for over 90% (0.63/0.68 = 0.926) of the autosomal recessive genetic load of non-Finnish Europeans.

Carrier detection probabilities calculated for unrelated couples are not valid for cases of consanguineous mating, who represent an important target group of screening programs. However, formula 1 can be extended easily to consanguineous couples by including their coefficient of relatedness, *r*, so as to read

2 $$1-{\prod }_{i=1}^{n}\left[1-\left[r{f}_{i}+(1-r){f}_{i}^{2}\right]\right]$$

Applying formula 2 to the 86 ACMG Tier 3 genes yields couple carrier detection probabilities that range from between 0.87 (EAS) and 3.71% (ASJ), for 2nd-degree cousins, to between 5.53 (EAS) and 10.67% (ASJ), for uncle-aunt or niece-nephew matings (Table 1). Carrier detection probabilities are the highest for consanguineous couples of ASJ ethnicity because the choice of the ACMG Tier 3 genes was influenced by several particularly high carrier frequencies in Ashkenazi Jews. For example, for 11 of the 19 genes with a maximum carrier frequency > 1/50 (Table 1 in Gregg et al. 2021), the respective ASJ frequency was the highest of all ancestries (NFE: 3, AFR:2, EAS: 2, SAS: 1, AMR: 0).

Assuming that the proportion of the genetic load attributable to the ACMG Tier 3 genes is the same for first cousins as for unrelated couples in non-Finnish Europeans (92.6%, see above), the incidence of autosomal recessive diseases among the offspring of first cousins should equal 6.37/4/0.926 = 17.2 in 1000 newborns. At first glance, this tenfold increase, compared to non-consanguineous couples, appears to contradict the widely held view that the relative risk of birth defects among the offspring of first cousins equals two (Stoltenberg et al. 1997). However, the incidence of (life) birth defects in the EU is currently estimated as 20.4 in 1000 newborns (https://eu-rd-platform.jrc.ec.europa.eu/eurocat/eurocat-data/prevalence_en), implying that the vast majority of cases, namely 2.04–0.17 = 1.87%, must be due to causes other than homozygosity or compound heterozygosity for recessive genetic variants. Since this surplus can be assumed to be unaffected by consanguinity, it should complement the incidence of autosomal recessive diseases for any mating type, including first cousins. This implies that the relative risk of births defects for the latter type should amount to (1.87% + 1.72%)/2.04% = 1.76 in non-Finnish Europeans, a figure not too far away from the reference value of two.

Additional evidence for the authenticity of the carrier detection probabilities listed in Table 1 for consanguineous matings comes from a population-based study by Abouelhoda et al. (2016). Based upon whole exome and targeted panel DNA sequencing of > 7000 individuals, these authors concluded that at least 0.7% of children born to first-cousin parents in Saudi Arabia are affected by an autosomal recessive disease. Whilst gnomAD does not provide carrier frequencies specifically for Saudi Arabia, these may nevertheless be surmised to be not too different from gnomAD estimates for SAS. Under this assumption, the projected risk for an autosomal recessive disease of first cousin offspring in Saudi Arabia equals 3.73%/4 = 0.93%, a figure that fits well with the authors’ notion that their empirical estimate of 0.7% is likely to represent a lower limit to the true disease risk.

We hope that the above considerations will aid in the process of implementation of carrier screening programs and provide additional support to reproductive counselling and to obtaining informed consent.
